# Introduction of an assessment toolkit associated with increased rate of DLB diagnosis

**DOI:** 10.1186/s13195-021-00786-8

**Published:** 2021-02-19

**Authors:** A. Surendranathan, J. Kane, A. Bentley, S. Barker, R. McNally, C. Bamford, J.-P. Taylor, A. Thomas, I. McKeith, D. Burn, J. T. O’Brien

**Affiliations:** 1 Department of Psychiatry, Cambridge Biomedical Campus, University of Cambridge School of Clinical Medicine, Box 189, Cambridge, CB2 0QQ UK; 2 Centre for Public Health, Queen’s University Belfast, Belfast, UK; 3 Institute of Neuroscience, Newcastle University, Newcastle upon Tyne, UK; 4 Population Health Sciences Institute, Newcastle University, Newcastle upon Tyne, UK

**Keywords:** Dementia with Lewy bodies, Parkinson’s disease dementia, Diagnosis

## Abstract

**Background:**

Dementia with Lewy bodies (DLB) and dementia in Parkinson’s disease (PDD) are recognised to be under-recognised in clinical practice in the UK, with only one third to a half of expected cases diagnosed. We aimed to assess whether clinical diagnostic rates could be increased by the introduction of a structured assessment toolkit for clinicians.

**Methods:**

We established baseline diagnostic rates for DLB and PDD in four memory clinics and three movement disorder/Parkinson’s disease (PD) clinics in two separate geographical regions in the UK. An assessment toolkit specifically developed to assist with the recognition and diagnosis of DLB and PDD was then introduced to the same clinical teams and diagnostic rates for DLB and PDD were reassessed.

For assessing DLB diagnosis, a total of 3820 case notes were reviewed before the introduction of the toolkit, and 2061 case notes reviewed after its introduction. For PDD diagnosis, a total of 1797 case notes were reviewed before the introduction of the toolkit and 3405 case notes after it. Mean values and proportions were analysed using Student’s *t* test for independent samples and *χ*^2^ test, respectively.

**Results:**

DLB was diagnosed in 4.6% of dementia cases prior to the introduction of the toolkit, and 6.2% of dementia cases afterwards, an absolute rise of 1.6%, equal to a 35% increase in the number of DLB cases diagnosed when using the toolkit (*χ*^2^ = 4.2, *P* = 0.041).

The number of PD patients diagnosed with PDD was not found overall to be significantly different when using the toolkit: 9.6% of PD cases before and 8.2% of cases after its introduction (*χ*^2^ = 1.8, *P* = 0.18), though the ages of PD patients assessed after the toolkit’s introduction were lower (73.9 years vs 80.0 years, *t* = 19.2, *p* < 0.001).

**Conclusion:**

Introduction of the assessment toolkit was associated with a significant increase in the rate of DLB diagnosis, suggesting that a structured means of assessing symptoms and clinical features associated with DLB can assist clinicians in recognising cases. The assessment toolkit did not alter the overall rate of PDD diagnosis, suggesting that alternate means may be required to improve the rate of diagnosis of dementia in Parkinson’s disease.

## Introduction

Lewy body dementia, consisting of dementia with Lewy bodies (DLB) and Parkinson’s disease dementia (PDD), is the second most common neurodegenerative dementia in older people and comprises 15–20% of dementia cases in pathological studies [1, 2].

However, the clinical prevalence of Lewy body dementia (LBD) is found to be much less, with systematic reviews reporting the prevalence of DLB to be 4.2–5% [3, 4] and PDD to be 3.6%, of all dementia cases [5]. In addition, our group investigated the diagnostic rate of DLB in clinical practice in two regions in the UK and found it to be 4.6% [6]; hence, DLB is likely to be under-diagnosed in these regions. Our group also found the proportion of Parkinson’s disease (PD) cases with dementia (i.e. PDD) was 9.7%, also below that expected when compared with the prevalence of between 20 and 30% reported in a systematic review [5]. We now sample a proportion of the same services included in the earlier report, assessing diagnostic rates before and after the introduction of an assessment toolkit designed to improve recognition and diagnosis of DLB and PDD.

Consensus diagnostic criteria for DLB [7, 8] and PDD [9, 10], aid clinicians in assessing patients with suspected Lewy body dementia. These criteria depend on eliciting a number of symptoms and clinical signs supported by the use of biomarkers, although a definitive diagnostic test is not yet available. DLB in particular is a heterogeneous condition with variable presenting features [11], often co-existing with significant Alzheimer’s pathology which can make diagnosis challenging, as core features may be masked leading to a diagnosis of AD [12, 13]. In a recent autopsy study, of the 30 cases with DLB (alone or mixed with AD), only 6 were correctly identified clinically as having DLB pathology, with the majority diagnosed as either AD or mixed AD and vascular dementia [14]. Delays in diagnosis can also occur, with higher attendances at clinic, a higher number of imaging tests required and more alternative diagnoses given to those with DLB than with other types of dementia [15, 16].

A diagnostic toolkit could provide a structured framework for the assessment of patients suspected of Lewy body dementia and might assist in raising the clinical diagnostic rate closer to the rates of LBD that are reported at autopsy. Whilst there are examples of structured assessment tools used in the diagnosis of dementia, the efficacy of such methods is yet to be assessed. In other medical disciplines, for example paediatrics and dermatology, toolkits have been found to be beneficial in diagnosis and in improving knowledge and behaviour when used as a diagnostic framework [17, 18].

Here we investigate whether the introduction of such a diagnostic toolkit to clinicians in secondary care improved the diagnostic rates of LBD.

## Methods

### Objectives

The primary objective of the study was to assess whether the introduction to NHS clinical services of a diagnostic toolkit would be associated with a change in the clinical diagnostic rates of DLB and PDD.

### Study design

We undertook a prospective study, examining prevalence rates for DLB and PDD diagnosis covering an 18-month period before and after the introduction of the assessment toolkit.

Services within two NHS Trusts in the North East and East Anglia of the UK, the same regions that were previously sampled for DLB and PDD diagnostic rates [6]. The services consisted of four memory clinics that were assessed for the rate of DLB diagnosis, these four services were a part of the nine services sampled in the previously performed prevalence study (Kane et al.) [6]. In addition, three movement disorders (or PD) clinics were assessed for the rate of diagnosis of PDD; similarly, these three services formed part of the five services sampled in the previously performed prevalence study.

The toolkit was based on the established diagnostic criteria for DLB and PDD [19, 20] and was developed following piloting in a service not involved in the current study. The toolkits set out the diagnostic criteria for PDD and DLB together with the questions to ask either the patient or carer to assess whether the patient fulfils such criteria. A systematic method for assessing for parkinsonism was also included. The entire toolkit and details of the pilot study can be found through the website link in the ‘Availability of data and materials’ section below.

The assessment toolkit was introduced to clinicians at an initial site visit, before a training session on how to apply the toolkit in clinical scenarios was provided by the study investigators, including where required teaching sessions on examining for parkinsonism. The study team provided further support on the use of the toolkit during the course of the study when requested by clinicians.

### Procedure

The notes of all subjects seen in these services were reviewed to identify patients with a diagnosis of dementia (for DLB prevalence), or those with a diagnosis of PD (for PDD prevalence), attending each participating service, for an 18-month period before the introduction of the toolkit and then an 18-month period after its introduction.

### Statistical analysis

Statistical analysis was performed using SPSS 26.0 for Windows. Confidence intervals for prevalence were calculated using the Wilson method. Mean values and proportions were analysed using Student’s *t* test for independent samples and *χ*^2^ test, respectively. For each test statistic, *p* < 0.05 was regarded as statistically significant.

## Results

### Demographics

In the DLB part of the study, from the period prior to the introduction of the toolkit, 3820 case notes were reviewed from memory clinics and 2140 dementia cases identified (consisting of 1460 in the North East and 680 in East Anglia). From the period after the introduction of the toolkit, 2058 case notes were reviewed from the same services and 1279 dementia cases detected (consisting of 632 in the North East and 647 in East Anglia).

There were no significant differences in age or sex between the groups assessed prior to the introduction of the toolkit and the group assessed after the introduction of the toolkit (see Table 1 - Demographics).

**Table 1:** Demographics

	Pre	Post	Statistic	***P***
**Age (mid screening period)**	Years (SD)	Years (SD)	*t* test	
DLB study	82.2 (7.9)	82.1 (7.5)	0.18	0.88
PDD study	80.0 (7.1)	73.9 (10.2)	19.2	< 0.001
**Sex**	Male/female (%male)	Male/female (%male)	*χ*^2^	
DLB study	865/1275 (40.4%)	499/780 (39.0%)	0.66	0.42
PDD study	666/461 (59.1%)	1233/734 (62.7%)	3.89	0.048

Age and sex comparison between subjects (pre and post introduction of the assessment toolkit) for both DLB and PDD parts of the study

In the PDD part of the study, from the period prior to the introduction of the toolkit 1797 case notes were reviewed from movement disorder or PD clinics across the two regions, with 1130 cases of Parkinson’s disease identified, consisting of 974 in the North East and 156 in East Anglia. In the 18-month period following the introduction of the toolkit, 3405 case notes were reviewed from the same services, with 1967 cases of PD identified, consisting of 1809 in the North East and 158 in East Anglia.

The group of PD cases sampled prior to the introduction of the toolkit was significantly older than the group which was sampled after the introduction of the toolkit, by a mean of 6.1 years (*P* < 0.001). This earlier group also had a slightly higher proportion of females (*p* = 0.048), see Table 1.

### Dementia with Lewy bodies

**Fig. 1:**
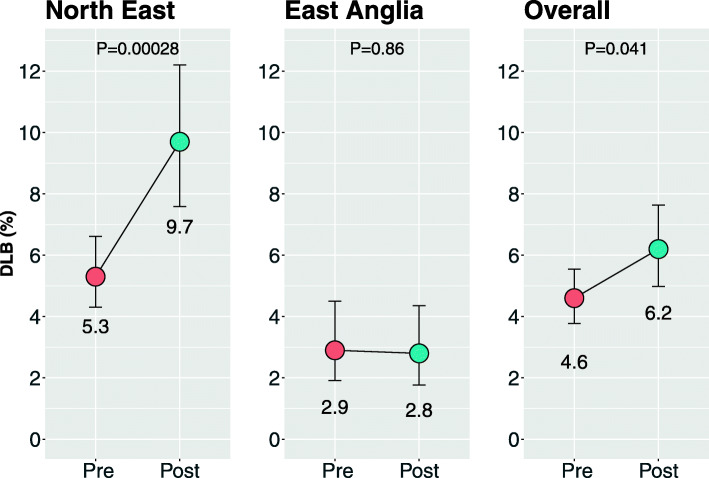
DLB diagnosis as a proportion of all dementia cases, before and after the introduction of an assessment toolkit for the diagnosis of DLB in the two regions sampled as well as overall (error bars represent 95% confidence intervals)

Before the introduction of the toolkit, the proportion of dementia cases diagnosed with DLB was found to be 4.6% (95% confidence interval (CI) 3.8% to 5.5%). Following the introduction of the toolkit, DLB was diagnosed in 6.2% (CI 5.0% to 7.6%) of dementia cases (see Fig. 1). This is an absolute rise of 1.6%, equal to a 35% increase in the cases diagnosed compared to the baseline (*χ*^2^ = 4.16, *P* = 0.041).

With respect to the different regions, in the North East, there was a significant increase from 5.3 to 9.7% (*χ*^2^ = 13.2, *P* < 0.001) after the toolkit was introduced. In East Anglia, however, there was no significant change, with a rate of 2.9% and 2.8% (*χ*^2^ = 0.30, *P* = 0.86), pre and post introduction of the toolkit, respectively.

**Fig. 2:**
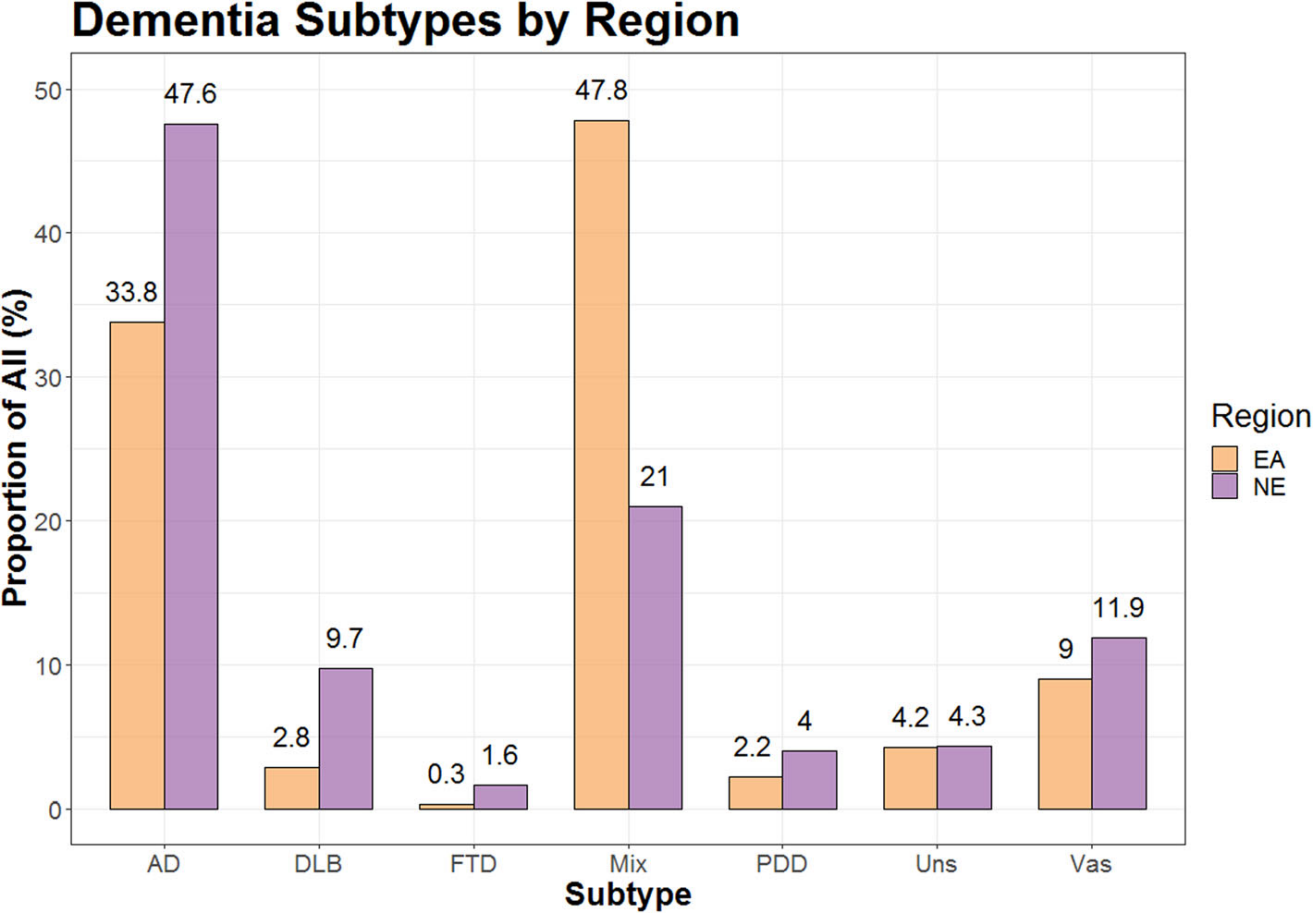
Dementia subtypes in the two regions after the introduction of the toolkit. AD, Alzheimer’s; FTD, fronto-temporal dementia; Mix, mixed dementias (excluding DLB); Uns, dementia subtype unspecified; Vas, vascular dementia

Since the rate of DLB diagnosed differentially increased between the regions, we looked at other diagnostic categories for dementia to see if this would shed insights into why the diagnostic rates increased in one region, but not the other. The diagnosis rates of the different subtypes as recorded within each region can be seen in Fig. 2.

Following the introduction of the toolkit, the diagnosis of dementia subtypes across both regions showed Alzheimer’s disease and mixed dementia as the commonest diagnosed across both regions, with Alzheimer’s recorded as having a higher rate in the North East and mixed dementia recorded higher in East Anglia.

### Parkinson’s disease dementia

**Fig. 3:**
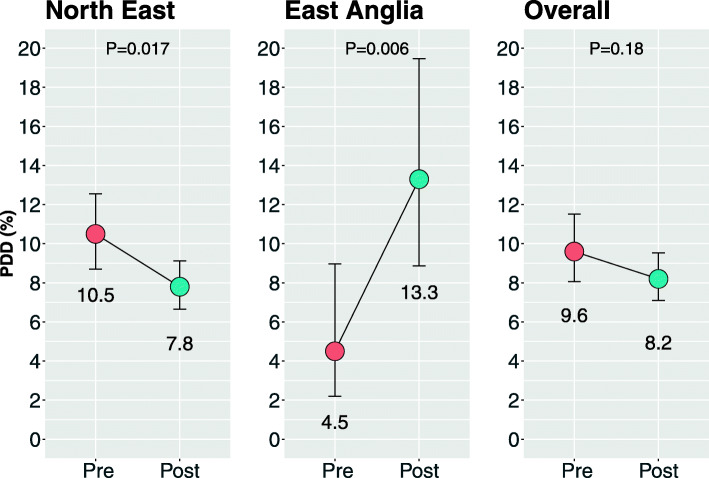
PDD diagnosis as a proportion of all PD cases, before and after the introduction of an assessment toolkit for the diagnosis of PDD in the two regions sampled as well as overall (error bars represent 95% confidence intervals)

In the initial survey, prior to the toolkits’ introduction, the number of PD patients diagnosed with PDD was found to be 9.6% (CI 8.1% to 11.5%). Following the introduction of the toolkit, PDD was diagnosed in 8.2% (CI 7.1% to 9.5%), which did not represent a statistically different change (*χ*^2^ = 1.79, *P* = 0.18)—see Fig. 3.

There were contrasting results in the different regions. In East Anglia, there was a significant increase from 4.5 to 13.3% (*χ*^2^ = 7.49, *P* = 0.006). In the North East, however, there was a significant decrease from 10.5 to 7.8% (*χ*^2^ = 5.7, *P* = 0.017).

### Comparison with earlier prevalence study

**Fig. 4:**
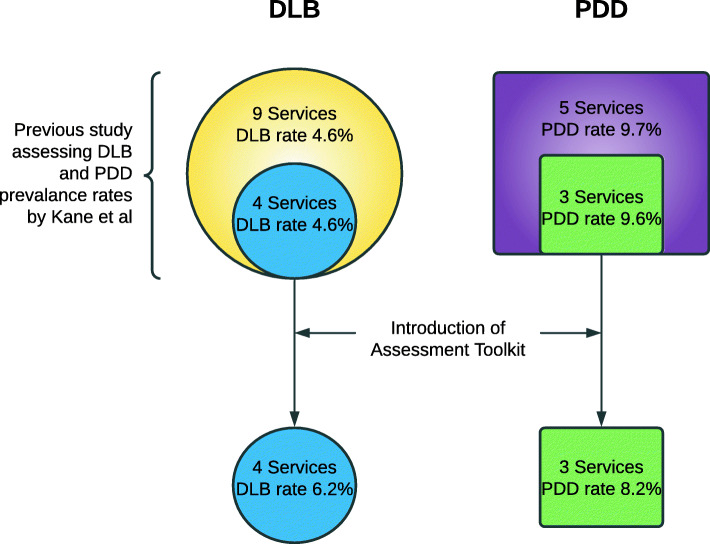
DLB and PDD rates pre and post introduction of the assessment toolkit compared to those reported in the earlier prevalence study [6]

A comparison of the results here with those from the earlier prevalence study shows that the services sampled in this study had similar DLB rates and PDD rates prior to the introduction of the assessment toolkit as the services sampled in the larger prevalence study [6] (see Fig. 4), with DLB and PDD rates the same or very similar to the larger samples.

## Discussion

### DLB diagnosis rates

The introduction of an assessment toolkit was associated with a significant increase in the rate of DLB diagnosis, suggesting that a structured means of assessing patients for DLB together with an increased awareness of DLB criteria during clinician assessment may increase the likelihood of diagnosis. This may reflect the heterogeneity of the condition at presentation, as core features may be obscured by other more troublesome clinical complaints such as psychiatric disturbance or falls. Yet direct questioning may reveal the underlying Lewy body disorder, with for example targeted questioning for rapid eye movement (REM) sleep behaviour disorder or fluctuating cognition. DLB is recognised as a challenging disorder to diagnose: DLB patients receive more alternative diagnoses and experience a longer length of time before receiving a final diagnosis [16]. Care-givers report the majority of patients see more than three doctors before a Lewy body diagnosis is made [15]. A DLB assessment toolkit may therefore be the support that clinicians need to help diagnose DLB quickly and accurately. In addition, a general increased knowledge and awareness of DLB following-on from the study team’s site visits and training sessions could also have contributed to the increase in diagnosis.

Despite the overall increase in diagnostic rates following the introduction of the assessment toolkit, there were regional differences found. In East Anglia, there was a no significant change (2.9% to 2.8%, *P* = 0.86) in diagnostic rates of DLB, whereas in the North East, there was an absolute increase of 4.4% (5.3% to 9.7%, *P* < 0.001).

Factors, such as a lower level of engagement in the toolkit, may have affected the outcome in East Anglia.

In addition, services in East Anglia recorded more dementia patients with a ‘mixed’ subtype (47.8%) than an Alzheimer’s dementia (AD) subtype (33.8%). A European wide systemic review of the prevalence of different dementia subtypes in those older than 65 found the prevalence of AD to be 53.7% [21]. A UK survey of dementia prevalence commissioned by the Alzheimer’s Society found AD to make up 62% of all dementia cases [22]. Both of these figures are higher than that reported for Alzheimer’s dementia in East Anglia. Yet 47.8% of dementia cases were found to be ‘mixed’ in East Anglia, and this excluded cases which specifically stated a combination of DLB with another subtype, which were simply recorded as DLB. This could mean some DLB cases were recorded as ‘mixed’ reflecting diagnostic uncertainty on the part of the clinician. This could also explain a lower rate of AD. Indeed the percentage of cases recorded as mixed was much higher than the 10% reported by the Alzheimer’s Society [22].

The higher increase of DLB diagnosis in this study was in the North East which also had the higher diagnosis rate before the introduction of a toolkit, suggesting the toolkit may not necessarily have the biggest benefit to services with the lowest rates. It may be that the toolkit enhances diagnostic performance in clinicians already aware of, and confident in, diagnosing DLB. Those less certain about diagnosis may require additional support and more extensive training in its use, rather than simply introducing it directly into practice.

These results also suggest that clinical prevalence may be higher than previously recorded. Systematic reviews report the prevalence of DLB to be 4.2–5% [3, 4], yet the levels detected in this study in the North East were 9.7% (CI 7.6% to 12.2%), nearing the rate of DLB seen in autopsy studies of 15% [2]. In the absence of a simple diagnostic test, a structured assessment toolkit may be what is required to improve detection of DLB, leading to the significant benefits of early and accurate diagnosis for patients and their carers.

Correctly diagnosing DLB is important for patients and their carers [23]. Failure to recognise autonomic symptoms secondary to the disorder such as orthostatic hypotension which can cause falls or bladder dysfunction that harshly affects independence and confidence can have severe effects on a patient’s quality of life [24]. The use of anti-psychotics can lead to worsening of an undiagnosed movement disorder and in severe cases can be fatal secondary to akinetic crisis [25]. Hence, improving detection will be significant for this group of patients.

### PDD diagnostic rates

In contrast to DLB, the assessment toolkit did not alter the overall rate of PDD diagnosis.

It was nevertheless notable that in East Anglia, there was a significant increase in PD dementia diagnosis rates following the introduction of the toolkit—an increase of 4.5% to 13.3%, or an absolute rise of 8.8%. This large rise, nearly trebling the rate of diagnosis, may indicate an assessment toolkit could assist in services where diagnosis rates are lower, as the rate in East Anglia (4.5%) was much lower than in the North East (10.5%) prior to its introduction. However, a limiting factor in this finding is the relatively small sample group in East Anglia, with only 158 PD patients compared with 1809 in the North East. The significant reduction in diagnostic rates within the larger group of PD patients in the North East nullified the large improvement in East Anglia and leads to the conclusion that there is no associated rate rise in PDD diagnosis from the introduction of the toolkit.

Reasons why no overall improvement in diagnosis was recorded may include clinicians’ level of engagement in the study and hence utilisation of the toolkit. A qualitative process evaluation on implementation of the toolkits will be reported separately. Another factor may be that the group surveyed following the introduction of the toolkit was younger (73.9 years) than the group studied in the initial pre-toolkit survey (80.0 years). It is well recognised that older age is associated with higher rates of dementia, including in PD [26].

In addition, Parkinson’s disease patients already have an established Lewy body disorder so the difficulties in the diagnosis of PDD are probably very different to that of differentiating DLB from other dementia subtypes. The diagnosis of PDD requires the recognition of a dementia syndrome, which can be facilitated by a cognitive assessment and a collateral history of functional decline. Notably, difficulties in integrating a formal cognitive assessment into an initial patient assessment were reported in the pilot trial of the toolkit [19]. Even when these are carried out, the diagnosing clinician may remain uncertain about the degree of severity of such impairments and also the degree to which they can be attributed to motor, rather than cognitive deficits. There may also be a reluctance to formally diagnose dementia [16] because of its stigmatising effect. None of these potential barriers to the diagnosis of dementia in PD is likely to be affected solely by the introduction of a structured assessment toolkit. An alternate means may be required to improve the rate of diagnosis of dementia in Parkinson’s disease.

### Limitations

The relatively small PD group size in East Anglia is a limitation of the study, meaning the large rise in diagnostic rate in this region is difficult to interpret. It is another limitation that only seven services were sampled in total. However, the diagnostic rates recorded prior to the introduction of the assessment toolkit were very similar to that seen in the larger prevalence study of 14 services [6], suggesting the services sampled here were a good reflection of that larger group.

## Conclusions

The introduction of an assessment toolkit was associated with a significant increase in the diagnosis of DLB, but this may be because clinicians already confident in diagnosing DLB are able to benefit from the introduction of a simple toolkit to reference. The high number of mixed dementia cases highlights diagnostic uncertainty amongst some clinicians, and those who are less confident in diagnosing DLB may gain confidence from greater training on the use of the toolkit and may lead to even higher diagnosis rates.

Contrasting changes in diagnosis rates of PDD were observed regionally following the introduction of the toolkit, which may be explained by the small samples size in East Anglia, but maybe also by the differing diagnostic process for diagnosing dementia in PD as compared to making a DLB subtype diagnosis.

## Data Availability

The datasets used and/or analysed during the current study are available from the corresponding author on reasonable request. The toolkits are freely available on the following website: https://research.ncl.ac.uk/media/sites/researchwebsites/diamond-lewy/Thomas_et_al-2018-International_Journal_of_Geriatric_Psychiatry.pdf

## Abbreviations

AD: Alzheimer’s dementia
DLB: Dementia with Lewy bodies
EA: East Anglia
LBD: Lewy body dementia
NE: North East
PD: Parkinson’s disease
PDD: Parkinson’s disease dementia
REM: Rapid eye movement
